# Automated Processes and Artificial Intelligence in Generating Candidates for Oncology Drug Repurposing: Three-Year Scoping Review of Data

**DOI:** 10.3390/pharmacy14040096

**Published:** 2026-07-01

**Authors:** Antonio Ivanov, Ines Hababa-Ivanova, Savina Elitova, Svetoslav Stoev, Violeta Getova-Kolarova

**Affiliations:** 1 Department of Organization and Economics of Pharmacy, Faculty of Pharmacy, Medical University-Sofia, 2, Dunav Str., 1000 Sofia, Bulgaria; antoniogivanov98@gmail.com (A.I.); ines.hababa98@gmail.com (I.H.-I.); selitova@gmail.com (S.E.); v.getova@pharmfac.mu-sofia.bg (V.G.-K.); 2 Department of Pharmaceutical Sciences and Social Pharmacy, Faculty of Pharmacy, Medical University-Pleven, 5800 Pleven, Bulgaria

**Keywords:** drug repurposing, drug candidates, artificial intelligence, big data, databases, RWE

## Abstract

Oncology conditions are increasingly defined by their molecular profiles, and drug repurposing exploits this new evidence to identify new therapeutic uses of authorized/investigational medicinal products outside their original indication(s). This scoping review mapped original research published between January 2022 and December 2024 to determine the impact of automated processes and artificial intelligence in generating oncology candidates for drug repositioning, and 42 individual projects met the eligibility criteria and were analyzed. The included studies demonstrate extensive use of computational approaches for candidate prioritization, large-scale data integration, and hypothesis generation in oncology drug repurposing, creating opportunities for positive impact on efficiency. The included projects most commonly were target-oriented and disease-oriented and used multiple databases and computational validation procedures, while experimental and clinical validation were less frequently reported. The available open-access literature suggests substantial activity in China and India, which can support the notion that digitalization represents an important instrument in healthcare systems of low- and middle-income countries but should be interpreted cautiously. While the search was limited to PubMed and open-access English-language publications, we identified a relatively small number of drug-oriented projects, the importance of providing publicly accessible source code to reduce development costs, and the predominant role of academic institutions.

## 1. Introduction

Drug repurposing (repositioning) is grounded in two principal scientific concepts: first, the discovery—following the human genome research—that certain diseases share common biological mechanisms and, secondly, the concept of pleiotropic medicines. Owing to advances in biomedical science, diseases today can be characterized through their molecular profiles (e.g., genes, biomarkers, signaling pathways, and environmental factors), which enables qualitative and quantitative assessment of similarity between different pathological conditions. In addition, numerous medicinal products with a long history of clinical use are phenotypically well characterized. This diversity of pharmacological effects arises from the pleiotropic capacity of drug molecules to interact with multiple biological targets (both primary and secondary). This underlies the possibility that a given medicinal product may be effective against different diseases, namely, when a secondary biological target of the drug is involved in the pathophysiology of another condition. Similar to diseases, drug molecules themselves may also be investigated for similarity, which can serve as the basis for initiating a repositioning process [1].

**Figure 1:**
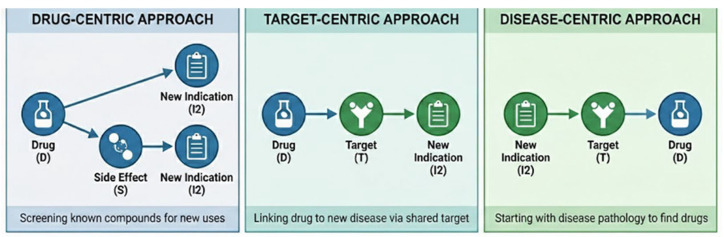
Types of repurposing according to their primary focus [3]. The figure is adapted from [3] and redesigned by the authors to reflect the scope of the present review.

Earlier repurposing efforts were often serendipitous, whereas contemporary approaches are increasingly systematic and data-driven. A more rational and systematic strategy in repositioning initiatives is used nowadays [2], where the interaction of a drug molecule (D) with one or more targets (T) produces several biological effects, which may be beneficial for a therapeutic indication (I) or may lead to adverse drug reactions (S). In disease-oriented drug repositioning, the clinical use of a medicine is expanded from its original indication (I) to a closely related indication (I2) (Figure 1). In target-oriented repurposing, identification of a new indication (I2) is linked to a well-established therapeutic target, whereas in drug-oriented repositioning, a new target (T2) is identified that connects the medicinal product to a novel indication [3].

At the core of repositioning lies a systematic understanding of molecule–target interactions. Experimental identification of such interaction properties is limited by low efficiency in both scope and throughput. This has led to the development of in silico methodologies that leverage computational power to detect the most potent molecule–target interactions [4]. The principal computational approaches include ligand-based, target-based, and machine-learning-based methods. Ligand-based approaches utilize information on candidate ligands and ligands within the target molecule to predict binding affinity; however, their performance is limited when few ligands are known for the target structure [5]. Target-based approaches are powerful tools for identifying spatial similarity between a drug and its molecular target, but they require full knowledge of the three-dimensional structures of both molecules, an especially challenging prerequisite for large receptors (such as G-protein-coupled receptors), or candidate molecules selected for repositioning. Incorporating artificial intelligence techniques (e.g., machine learning) into these processes enables faster and more efficient detection of molecule–target similarity. This is achieved by integrating database information on interactions between diverse ligands, grounded in the principle that structurally similar ligands tend to interact with similar proteins. This way, the need to fix one side of the process (drug molecule or target) is eliminated, allowing simultaneous analysis of vast numbers of compound combinations. The most widely used machine learning approaches include matrix-factorization-based, network-based, and kernel-based models. Despite their advantages over other strategies, these approaches require structured and comprehensive databases, which are not always available; moreover, information on dose–response relationships or other key parameters may be lacking [6,7].

One of the most prominent ligand-based approaches is quantitative structure–activity relationship (QSAR) analysis, which aims to identify statistically significant correlations between chemical structure and biological or toxicological properties expressed either parametrically (pIC50, pEC50, and Ki) or qualitatively (active/inactive and toxic/non-toxic), using regression or classification techniques, respectively [8]. Among target-based approaches, molecular docking is the most widely applied method: it predicts with high precision the conformation and binding of ligands within the active site of a target molecule. Critical to this technique is determination of the spatial orientation at which ligand–target binding results in the lowest binding energy and strongest intermolecular interactions, thereby forming a stable ligand–target complex [9]. An illustrative example of machine learning approaches is the use of large-scale biomedical datasets, such as electronic health records, post-marketing surveillance data, and clinical trial databases. The availability of extensive, heterogeneous clinical information facilitates integrated analyses combining disease phenotypes, genetic or laboratory data, and drug exposure, where adverse event reports may provide evidence that a given medicinal product modifies a particular phenotype. Clinical trial datasets may also contain information that is not analyzed because it lies outside the primary hypothesis, and machine learning methods can reveal trends within specific subpopulations and link them to demographic, genetic, or clinical characteristics, thereby generating hypotheses for novel pharmacological effects of the investigational product. Furthermore, additional data may be accumulated through study protocols requiring the collection of biological samples and participant consent for future research use. Alongside the inherent limitations of database completeness and data quality, ethical and personal-data-protection considerations become particularly salient when handling patient-level information [10,11].

The use of pre-existing data also introduces further challenges for drug repurposing. One such challenge is the accuracy and consistency of analytical models applied to databases, as the use of different computational tools may generate variability and uncertain reliability. Another characteristic limitation is the heterogeneity of databases, which renders data structuring a slow and difficult process and highlights the need for standardized information clusters [12]. Quality control of both the data and the methods through which they were collected represents an additional constraint in database-driven repositioning research, as does the need for common standards to facilitate comparison of study outcomes [13]. Information contained in so-called “raw” databases may be of lower quality than that in experimentally curated datasets, as the former often rely on automated literature screening rather than multistage validation processes. Discontinuation of database updates also poses a risk to data quality when such resources are used to support hypothesis testing [14].

Despite the rapid expansion of computational approaches in drug repurposing, the evidence base remains fragmented with respect to oncology-specific applications, types of repurposing strategies, validation practices, and code availability. In particular, it is still unclear how often automated and AI-based pipelines progress beyond in silico prioritization toward experimental or real-world confirmation. A focused mapping of recent oncology repurposing studies is therefore needed to characterize the methodological landscape and identify the main translational gaps. The objective of this scoping review was to systematically map original research on automated processes and artificial intelligence used to generate oncology drug-repurposing candidates, with emphasis on strategy type, data sources, algorithms, validation level, code availability, and geographical distribution. In addition, the review seeks to examine patterns with particular attention to the role of academic and nonprofit organizations. By synthesizing this evidence, the review provides an overview of the current landscape, highlights methodological strengths and limitations, and identifies gaps for future research and development in AI-driven drug repurposing.

## 2. Materials and Methods

We performed a scoping review on 21 April 2026, using the PubMed database. This review was performed in accordance with the PRISMA (Preferred Reporting Items for Scoping Reviews and Meta-Analyses) guidelines, and the PRISMA-ScR checklist can be accessed in the Supplementary Material Section [15]. The source was selected owing to its widespread use in scientific research, free and open access, and extensive volume of available literature. In addition, this database allows for identification of interdisciplinary approaches from chemistry- to pharmacy-focused. To limit the search results, the following PubMed query was applied:

“(drug repurposing OR drug repositioning OR drug rescuing OR drug reprofiling) AND (artificial intelligence OR machine learning OR natural language processing) AND (oncology)”

This combination was selected to comprehensively capture the most commonly used synonyms for drug repurposing, to direct the search toward subfields of artificial intelligence frequently applied in healthcare, and to focus on oncology as a discipline characterized by a high concentration of unmet medical needs. In addition, the following eligibility criteria were applied:

- Inclusion criteria: scientific publications reporting original research projects related to drug repurposing, published in English between January 2022 and December 2024 and available via open access to ensure transparency and reproducibility of data extraction and methodological assessment.
- Exclusion criteria: review articles, discussion papers, communications, conference abstracts, editorials, and other non-original publication types.

Articles retrieved using the specified search terms and criteria were first subjected to an initial title and abstract screening to further refine application of the inclusion and exclusion criteria. Subsequently, all eligible publications underwent full-text analysis, and key characteristics relevant to the review were extracted using a structured data charting form: first author, country of origin, repurposing strategy, therapeutic focus, databases used, algorithmic approach, validation level, funding statement/authors’ affiliations, and code availability. Data charting was performed independently and then checked through discussion to ensure consistency. All authors contributed to each screening phase, and in cases where two or more authors classified a study differently, inclusion or exclusion was determined following a consensus discussion.

## 3. Results

**Figure 2:**
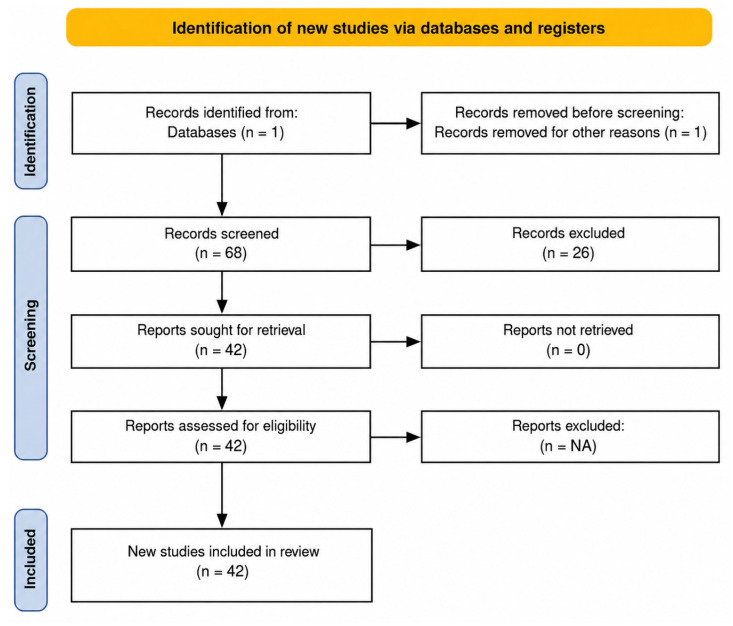
Scoping review flowchart.

A total of 42 studies meeting the predefined eligibility criteria were included in the analysis (Figure 2 and Appendix A). Across these studies, a consistent pattern emerges in the application of artificial intelligence (AI) to oncology drug repurposing, particularly in terms of output generation, methodological design, and data integration strategies.

### 3.1. Landscape of Study Selection and Geographic Origin

A total of 42 individual drug-repurposing projects were analyzed, representing a significant expansion in the utilization of automated pipelines and artificial intelligence (AI) within the last year. China (35.7%) represented the largest share of included open-access PubMed-indexed studies in this dataset, followed by India (9.5%) and the USA (7.1%); however, this should be interpreted as a descriptive finding rather than a measure of true global research intensity because the analysis was limited by the search strategy, database selection, and open-access restriction.

### 3.2. Strategy Types and Therapeutic Focus

For the purposes of this review, target-oriented studies were defined as those primarily aimed at identifying new ligands or binders for established molecular targets; disease-oriented studies were those matching drug effects to disease signatures or phenotypes; drug-oriented studies were those seeking new targets for known compounds. The analyzed projects showed the following distribution: target-oriented (45.2%), disease-oriented (38.1%), and drug-oriented (16.7%). Target-oriented studies largely utilized molecular docking and tensor decomposition to identify new binders for established oncogenic proteins. Disease-oriented approaches frequently employed transcriptomic reversal scoring (sRGES) to match drug-induced gene expression profiles against disease signatures.

Multi-cancer (pan-cancer) analysis was the most frequent indication (45.2% of studies), emphasizing the ability of AI to identify broad-spectrum therapeutic candidates across diverse tumor types. Specific focus areas included high-fatality or rare cancers such as glioblastoma, hepatocellular carcinoma, and diffuse intrinsic pontine glioma (DIPG).

### 3.3. Algorithmic Diversity and Data Integration

The complexity of algorithms has evolved from traditional machine learning (ML) to advanced deep learning (DL) architectures. Key methodologies included:

- Graph neural networks (GNNs): used for complex drug–target–disease association mapping.
- Large language models (LLMs): recently integrated for target identification and evidence synthesis, specifically using GPT-4 in prostate cancer research.
- Explainable AI (XAI): introduced to address the “black box” nature of deep learning, providing logic-based reasoning for drug–disease associations.

Data integration was robust, with studies utilizing an average of eight-plus databases. The most frequent sources included TCGA (The Cancer Genome Atlas), GEO (Gene Expression Omnibus), and DrugBank.

### 3.4. Levels of Validation

Validation was grouped into computational validation only, experimental validation, and real-world evidence or clinical-data-based validation. While computational validation remains the primary endpoint (35.7% of projects), there is an increasing trend toward higher-level evidence. In total, 11.9% of studies progressed to in vitro and in vivo experiments using animal models or cell viability assays, while only 7.1% of results utilized real-world evidence (RWE) like electronic health records (EHR) and claims data to emulate clinical trials, providing a direct bridge to clinical observation.

### 3.5. Transparency and Reproducibility

In total, 60% of studies (*n* = 25) provided publicly accessible source code, while 5% (*n* = 2) provided code upon request. This indicates a relatively high level of methodological transparency compared with traditional drug discovery approaches. In addition, a trend for broader programming code sharing can be observed comparing the start and end years of the study period.

## 4. Discussion

### 4.1. Efficiency and Output Patterns of AI-Driven Repurposing

The synthesized evidence indicates that AI-based repurposing frameworks consistently generate a high number of candidate molecules while reducing reliance on resource-intensive experimental screening. This efficiency appears to arise from three converging factors:

- Scalable computation.
- Integration of heterogeneous datasets.
- Parallel evaluation of multiple drug–target relationships.

The included studies suggest that computational approaches may facilitate large-scale candidate prioritization and integration of heterogeneous datasets. However, the present review did not identify standardized quantitative measures that would allow direct comparison of efficiency, cost, development time, or candidate yield between AI-based and conventional repurposing approaches. Choundhury et al. share the example that traditional methods relying on in vitro high-throughput screening involve large investments and sophisticated experimental setups, where AI/ML-aided methods can be used to identify initial strong candidates, which is a powerful option to save resources and time [16]. Although the development, training, and validation of artificial-intelligence-based methodologies are also time-consuming, subsequent computation and candidate generation occur rapidly owing to the high computational capacity of modern systems. Moreover, the availability of publicly accessible source code in some projects indicates a shift toward reproducible and collaborative research models, which can facilitate model reuse across indications, reduced development costs, and acceleration of discovery cycles. This could potentially reduce or even eliminate additional time and financial costs [17]. One of the projects included in the analysis (RepurposeDrugs) is even available as a public web platform, which allows not only monitoring of newly identified therapeutic applications, but also exploration of specific drug–indication relationships [18]. In their paper, Allarakhia describes the connections between information and communication-technology platforms, which facilitate open sharing of resources, technology, and intellectual property. Sharing facilities and joint program development to foster drug-repurposing human-capacity development are paramount for the cost- and time-effective advancement of new chemical entities targeting rare and neglected diseases [19]. However, the absence of standardized benchmarking can limit comparability between models. On this basis, it could be concluded that traditional experimental repurposing demonstrates markedly lower efficiency compared with contemporary digital approaches. However, high-output volume usually does not directly translate into clinical relevance, as most studies lack downstream validation.

### 4.2. Methodological Trade-Offs Across Repurposing Strategies

A divergence can be observed between repurposing strategies. Target- and disease-oriented approaches demonstrate higher throughput and broader candidate generation. Additionally, Khataniar et al. show that target-oriented strategies have a better chance of uncovering valuable leads as these take minimal time to complete the entire screening procedure and involve connecting a drug to a particular disease predicated on its target proteins, opposite to the polypharmacological techniques of the drug-oriented ones. In addition, pathway- or network-based techniques that leverage illness omics data, existing signaling or biochemical functions, and interacting protein connections to reassemble disease-specific circuits are presented to serve as key players in repositioning [20]. Drug-oriented approaches, while producing fewer candidates, may offer greater mechanistic novelty but require more detailed structural data. Findings from drug-oriented projects further reveal this mechanism to be less efficient than target-oriented or disease-oriented strategies. In their review, Parisi and colleagues likewise confirm the limited number of outputs from drug-oriented projects, noting greater innovation and specificity as strengths, but also highlighting the major challenge posed by the need for detailed crystal structures to enable accurate modelling of molecular interactions. The authors further emphasize the need to allocate additional resources to this form of repurposing, given that the other two approaches tend to identify new applications of medicinal products in indications closely related to the original one, which do not substantially contribute to reducing unmet medical needs [3]. This suggests a trade-off between efficiency (quantity) and biological specificity (quality). The dominance of target- and disease-based methods reflects their lower technical barriers and compatibility with existing datasets.

### 4.3. Central Role of Data Integration

**Figure 3:**
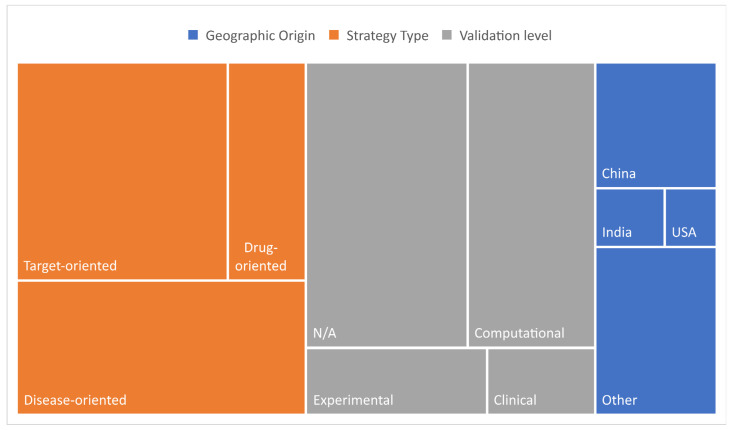
Main review results.

**Figure 4:**
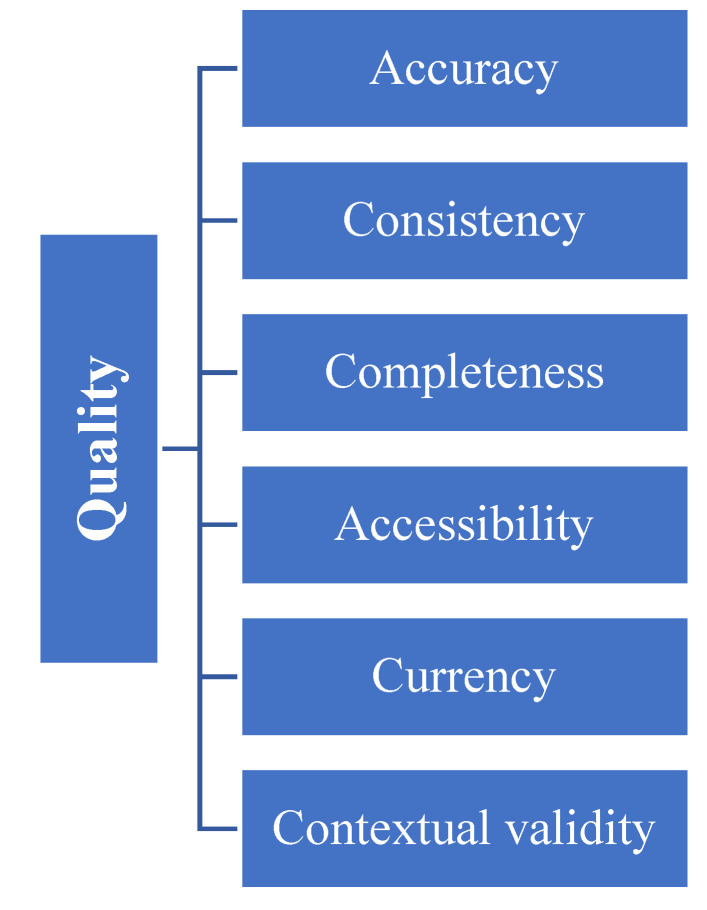
Categories for database quality assessment [23]. The figure was designed by the authors based on information reported in [23].

The consistent use of multiple databases across all studies suggests that data integration is a foundational component of AI-driven repurposing. However, this introduces systemic challenges like variability in data quality, lack of standardization, and potential propagation of bias. Despite high rates of methodological validation, there is a systematic lack of experimental and clinical validation. This represents a critical bottleneck where most models remain hypothesis-generating tools and very few progress to biological confirmation or clinical application. This gap limits the practical impact of AI-generated candidates and can contribute to the persistence of the “valley of death” in drug development. The extensive use of multiple heterogeneous databases is driven by the need to extract diverse information relating to diseases and/or targets and/or medicinal products—data that are seldom stored within a single source. It may be noted that, with the exception of DrugBank, which was established as a private initiative, most resources are developed and maintained by governmental (most commonly affiliated with the National Institute of Health) and/or academic institutions. Conversely, several of the analyzed studies also report the use of datasets compiled within the work of other research groups or smaller databases lacking institutional support and not included in established reference lists. This creates potential risks related to unreliable data quality and the generation of misleading conclusions [21]. As part of its mission to monitor data resources in the biological and health sciences, the Global Biodata Coalition recommends prioritizing databases characterized by high scientific quality, robust information–lifecycle management frameworks, interoperability with other data sources, and related safeguards [22]. The scientific community likewise converges around the need for demonstrable quality standards, with a number of authors proposing key evaluation parameters. Syed and colleagues define six categories (Figure 3 and Figure 4).

Although most of the articles validate their repurposing methodology, only a small number of the projects contain experimental validation (new lab or in vivo experiments performed by the authors) and experimental confirmation of translational relevance (e.g., functional biological validation, patient cohorts, and therapy-response evidence). In its project on strengthening digital competencies and examining the use of artificial intelligence in healthcare, the National Health Service (NHS) highlights the importance of validation. To enable ongoing monitoring and confirmation of technological performance, the following forms of validation are described: [24]

1. Internal validation: testing using a separate dataset, often derived from the original training source. Such validation is generally conducted using retrospective data.
2. External validation: testing using datasets that are not part of the original training source. This assesses whether the performance demonstrated during internal validation is maintained and evaluates the generalizability of the model. External validation may be conducted either by the developer or an independent evaluator.
3. Local validation: in some cases, large-scale external validation may be avoided, and local validation may instead be applied to ensure appropriate functioning within a restricted dataset.
4. Prospective validation using clinical-study data: real-time testing using real-world data. This approach can also assess whether the technology provides advantages in prediction and data analysis.
5. Ongoing performance monitoring: necessary to track effectiveness over time in response to changes in population characteristics, technical parameters, or other influencing factors.

Validation practices appear to function as a compensatory mechanism for these limitations, although they are predominantly computational rather than experimental.

In addition to technological validation, the effective translation of AI-generated drug-repurposing candidates into clinical practice is significantly reliant on academic leadership and the development of specialized capabilities. Academic institutions have always been pivotal in formulating repurposing methodology, creating collaborative research networks, and closing the translational divide between computational discovery and clinical application. Simultaneously, specialized educational programs, such as the REMEDi4ALL Drug Repurposing Bootcamp for Academics, seek to enhance methodological proficiency and expedite the translation of repurposing research into clinical applications through interdisciplinary training and collaboration [25,26].

### 4.4. Algorithmic Convergence Toward Hybrid Models

The findings reveal trends toward multi-algorithm frameworks, where different AI techniques are combined within a single pipeline. Four broad paradigms can be identified:

1. Systems-level models (network-based and tensor-based).
2. Classical machine learning models (e.g., SVM and random forests).
3. Phenotype- and single-cell-aware models.
4. Hybrid AI–experimental pipelines.

Among these, hybrid models demonstrate the strongest translational relevance but are least common due to higher resource requirements. Taguchi, Yeh, and Zhong use systems-level association models (tensor decomposition, graph-based representations, or network embedding), which emphasize global structure and pattern discovery but can be agnostic to causality. Karampuri, Sarker, Adamek, and López-Cortés utilize classical machine learning and ensemble models (e.g., random forests, deep neural networks, and hybrid ensembles) optimized for accuracy on held-out datasets, often treating biological entities as interchangeable features. Liu, Ianevski, and Jabarin work with single-cell and phenotype-aware AI frameworks, which explicitly capture heterogeneity and context specificity, a key advantage over bulk-omics AI, with additional strength being better alignment with biological reality models. On the other hand, the main limitation is that translational interpretation depends heavily on downstream validation. Zhang, Chen, Kim, Huang, and especially Lee use hybrid AI–experimental pipelines, in which they integrate AI predictions with wet-lab or human data validation where AI is used as a prioritization engine, not as a stand-alone discovery endpoint. This creates strong translational credibility but lowers the throughput and narrows the scope [27,28].

Although only a small number of studies identified in this review explicitly incorporated large language models (LLMs), their emergence represents an important recent development in AI-assisted drug discovery and repurposing. Unlike traditional machine learning approaches that primarily analyze structured datasets, LLMs are capable of processing and synthesizing large volumes of unstructured biomedical literature, clinical reports, patents, and database records. This capability may facilitate target identification, hypothesis generation, evidence summarization, and prioritization of candidate drug–disease relationships. However, one of the principal concerns is hallucination, whereby models generate plausible but factually incorrect statements, references, or biological relationships. Such outputs may be difficult to detect without extensive domain-specific validation and may introduce false hypotheses into the repurposing pipeline. Another challenge relates to knowledge grounding. The reliability of LLM outputs depends heavily on the quality, completeness, and recency of the underlying training data.

The swift integration of generative artificial intelligence is accompanied by significant global investment and deployment initiatives. China’s national AI programs have facilitated the incorporation of artificial intelligence into healthcare systems and biomedical research, whereas India has prioritized AI-driven public health innovation through partnership among academia, healthcare providers, and industry. Comparable advancements are increasingly seen in oncology medication repurposing and precision medicine, where artificial intelligence is emerging as a crucial element of translational research processes [29,30,31]. In rapidly evolving biomedical domains, model outputs may not reflect the most current evidence and may inadvertently reproduce biases present in published literature.

Future applications may benefit from retrieval-augmented generation architectures, integration with curated biomedical databases, and hybrid AI frameworks combining LLM-based knowledge extraction with network analysis, molecular modelling, and experimental validation. Such approaches may improve transparency, reduce hallucination risk, and enhance the translational utility of LLM-assisted drug-repurposing workflows [32].

### 4.5. Structural Role of Academia

While academia drives innovation, limited industry participation may hinder large-scale translation and commercialization. Among the 42 studies included in the review, only one project was developed by a pharmaceutical company (Lee et al.), while all remaining projects originated from academic institutions. This once again underscores the substantial leadership role of academia in drug repurposing, as well as the need for additional support to facilitate translation toward final outcomes. As previously said, academia serves as a primary catalyst for drug repurposing. In 2011, Oprea and colleagues reported the increasing academic involvement in drug repurposing, while at the same time warning of the risk of entering the so-called “valley of death” following initial progress, where scientific advances stagnate in the absence of sufficient regulatory support [25].

In response, alongside the establishment of supportive mechanisms by EMA and FDA, numerous conferences have been organized addressing the role of academia in repurposing, including RExPO (REPO4EU), the International Drug Repurposing Conference (REMEDI4ALL), and the Annual Drug Repositioning and Repurposing Conference (Arrowhead Publishers), among others. The REMEDi4ALL initiative has further strengthened this academic ecosystem by developing a specialized intensive training program in 2025 for researchers focusing on the complete pathway of drug repurposing and supporting the translation of hypotheses into accessible therapies [26]. The substantial contribution of academic institutions suggests that AI-driven repurposing is concentrated around academia, with less involvement from industry. This may reflect publication bias (industry confidentiality) or early-stage nature of AI-repurposing research.

The geographical distribution of projects observed in this review indicates that a substantial proportion of the included open-access publications originated from China. While previous literature highlights national AI strategies and digital health initiatives in these countries, caution is warranted in drawing causal inferences from the present dataset. Given the narrow temporal scope (single year), reliance on a single database (PubMed), and restriction to open-access publications, the observed distribution may reflect publication practices, indexing patterns, and funding mandates rather than actual global research intensity. Therefore, our findings should be interpreted as descriptive rather than explanatory. Li and colleagues emphasize the strategic relevance of AI in the People’s Republic of China, citing limited healthcare resources and the potential for improvement through both virtual tools and physical technologies, such as robotics. They further note that this development follows a deliberate national policy direction, exemplified by the AI development strategy issued by the State Council in 2017 [29]. Gore and colleagues describe the evolution and challenges of AI in the Republic of India, identifying innovation promotion and collaboration between institutions, academia, and the private sector as central drivers of digital transformation in healthcare, while also underscoring the critical importance of database development and governance as a starting point for digitalization [30]. Despite possessing substantial technological capacity and advancement in AI, the United States and EU member states were represented by only two projects. This may be partly explained by greater accessibility and coverage of healthcare services, which reduces the urgency of accelerated digitalization, as well as by strict data-protection requirements that represent additional barriers [31]. Future bibliometric analyses incorporating multiple databases (e.g., Scopus and Web of Science), subscription-based journals, and longer time horizons would be required to determine whether the observed geographical pattern reflects a sustained structural trend in AI-driven drug-repurposing research.

As AI-based models enable the integration of multiple algorithms within a single analytical framework, they also demonstrate exceptionally high combined efficiency. This is reflected in their capacity to process heterogeneous databases, reduce time and financial costs, enable model reuse and sharing across research teams, and generate multiple valuable outputs.

As a strength of our work, we consider the discussion on the importance of methodology validation due to the large quantity of utilized algorithms and databases and limited real-world evidence confirmation, as well as the findings of project distribution by country.

Within the present scoping review, certain limitations should be acknowledged as well, including reliance on a single database (PubMed). Although PubMed provides extensive coverage of biomedical research, it does not comprehensively index computer science, bioinformatics, engineering, or artificial intelligence venues. Consequently, methodological studies published in sources such as IEEE Xplore, ACM Digital Library, arXiv, Scopus, or Web of Science may have been underrepresented. Future reviews should incorporate multiple bibliographic databases to provide a more comprehensive assessment of algorithmic diversity and methodological innovation in AI-driven drug repurposing. In addition, the exclusion of review articles as a publication type, as well as the elimination of certain records following abstract-level screening, may also represent sources of limitation for the study.

The review was not designed to evaluate comparative efficiency outcomes. Most included studies focused on methodological development and candidate generation rather than reporting standardized metrics such as computational costs, screening duration, hit rates, or translational success rates. Consequently, quantitative conclusions regarding the superiority of AI-based approaches cannot be drawn from the available evidence.

The discussion on the leadership role of academia and nonprofit organizations in the whole repurposing process and in the implementation of automated processes and AI in particular can be considered both as a strength (we reconfirm the popular opinion of importance of regulatory agencies and scholarship) and a limitation (industrial research is often not disclosed through publications and is not suitable for literature-based analyses).

An additional important limitation arises from the restriction to open-access publications. Open-access status is not a neutral proxy for scientific activity, as publication models vary across countries, institutions, and funding frameworks. Industrial research and translational medicinal chemistry projects are frequently published in subscription-based journals or may remain unpublished due to intellectual property considerations. As a result, the relatively small number of included studies and their geographical distribution may partially reflect differences in publication accessibility rather than the true distribution of AI-driven drug-repurposing research. This limitation should be considered when interpreting both the quantitative findings and the country-level analysis.

## 5. Conclusions

Oncology drug repurposing likewise benefits from advances in computational technologies through the introduction of algorithms such as artificial intelligence. The available open-access literature indicates their application enables large-scale integration of heterogeneous datasets and systematic generation of repurposing hypotheses, although quantitative comparisons with conventional approaches remain limited. It is further observed that the majority of the identified repurposing projects incorporating artificial intelligence employ multiple databases, provide publicly accessible source code, and implement methodological validation procedures. The leading representation of China and India in the retrieved open-access literature suggests active engagement in AI-driven oncology drug repurposing; however, this observation should be interpreted cautiously, as publication accessibility models and database selection may have influenced the apparent geographical distribution. The review also identifies limitations in the number of drug-oriented repurposing projects, as well as the predominant role of academic institutions in driving this therapeutic development strategy.

## 6. Future Directions

As authors, we believe that automated processes and artificial intelligence (AI) are increasingly central to oncology drug repurposing, yet their translational impact remains constrained by limited biological and clinical integration. Future research should prioritize hybrid computational–experimental frameworks, in which AI-driven predictions are iteratively refined through functional validation using high-throughput cellular assays, organoids, or perturbation screens. Such closed-loop systems can reduce false discovery rates and improve biological relevance.

Advances in multimodal data integration will be critical to capturing the complexity of cancer biology. Integrating genomic, transcriptomic, proteomic, metabolomic, and imaging data through modern deep learning architectures may enable more accurate modeling of drug mechanisms and tumor heterogeneity. In parallel, incorporation of single-cell and spatially resolved data can reveal cell-state-specific vulnerabilities and microenvironment-dependent drug responses, supporting more precise repurposing strategies.

To facilitate clinical adoption, future AI systems must emphasize explainability and mechanistic interpretability, enabling transparent links between predicted candidates and underlying biological processes. Context-aware drug-ranking models that incorporate clinical outcome data and real-world evidence may further enhance translational relevance and patient specificity.

Finally, broader adoption of privacy-preserving learning frameworks, standardized benchmarking datasets, and early engagement with regulatory stakeholders will be essential to ensure robustness, reproducibility, and regulatory readiness of AI-generated repurposing candidates. Collectively, these advances can accelerate the translation of automated AI frameworks into clinically actionable oncology therapies.

## Data Availability

No new data were created or analyzed in this study. Data sharing is not applicable to this article.

## Supplementary Materials

The following supporting information can be downloaded at: https://www.mdpi.com/article/10.3390/pharmacy14040096/s1, File S1: Preferred Reporting Items for Systematic reviews and Meta-Analyses extension for Scoping Reviews (PRISMA-ScR) Checklist.

## Abbreviations

The following abbreviations are used in this manuscript:

AI	Artificial intelligence
EU	European Union
QSAR	Quantitative structure–activity relationship
GEO	Gene Expression Omnibus
GO	Gene Ontology
TCGA	The Cancer Genome Atlas
SVM	Support vector machines
XGBoost	Extreme Gradient Boosting
NHS	National Health Service

## Appendix A

### Publications Included in the Scoping Review

**DOI**	**First** **Author**	**Code Availability**
10.1186/s12859-024-06009-9	Taguchi	https://github.com/tagtag/TDbasedUFEPPI (accessed on 4 January 2026)
10.3389/fimmu.2024.1497300	Zhang	https://github.com/Zaoqu-Liu/IRLS (accessed on 4 January 2026)
10.1038/s41598-024-79391-2	Sarker	Not available
10.1016/j.compbiomed.2024.109158	Adamek	https://github.com/Sanofi-Public/indication-finding (accessed on 4 January 2026)
10.1093/gpbjnl/qzae036	Zhong	https://github.com/weilin-genomics/rSuperFeat (accessed on 4 January 2026)
10.3390/ijms25105267	Xu	Not available
10.1016/j.phrs.2024.107082	Huang	Not available
10.1111/jcmm.70085	Chen	https://github.com/CHR991004/AML_Pipeline (accessed on 4 January 2026)
10.1038/s41598-024-71076-0	Karampuri	https://github.com/ecrl/padelpy (accessed on 4 January 2026)
10.1093/bib/bbae490	Liu	https://github.com/Chonghui-Liu/DrugReSC (accessed on 4 January 2026)
10.1093/gpbjnl/qzad008	Yeh	https://github.com/Bin-Chen-Lab/transcell (accessed on 4 January 2026)
10.1038/s41598-024-68565-7	López-Cortés	https://github.com/muntisa/machine-learning-for-druggable-proteins (accessed on 4 January 2026)
10.1371/journal.pone.0307649	Mohammadzadeh-Vardin	https://git.dml.ir/taha.mohammadzadeh/DeepDRA (accessed on 4 January 2026)
10.1093/bib/bbae328	Ianevski	https://github.com/IanevskiAleksandr/repurposedrugs (accessed on 4 January 2026)
10.1038/s41467-024-49620-3	Tong	https://github.com/myzhengSIMM/TranSiGen (accessed on 4 January 2026)
10.1371/journal.pcbi.1011851	Chen	https://github.com/codejiajia/AutoEdge-CCP (accessed on 4 January 2026)
10.3390/ijms25179392	Baser	Not available
10.1093/bib/bbae108	Jabarin	https://github.com/eyalmazuz/DrugRepurposing (accessed on 4 January 2026)
10.3390/ijms25020765	Kim	Not available
10.1038/s41591-024-03224-y	Lee	https://github.com/RebekkaWegmann/drugseq_toolbox (accessed on 4 January 2026)
10.1038/s41467-022-34277-7	Chen	https://github.com/OSU-BMBL/scDEAL (accessed on 4 January 2026)
10.1038/s41467-022-28523-1	Ravi	https://github.com/heilandd/Vis_Lab1.5 (accessed on 4 January 2026)
10.1016/j.tranon.2023.101741	Zhang	Not available
10.3389/fphar.2022.936758	Huang	https://github.com/Clement-HUANG/LUAD-Drug-Repositioning (accessed on 4 January 2026)
10.3389/fimmu.2022.977358	Gimeno	Not available
10.1016/j.bmc.2022.117043	Rang	Not available
10.3390/ijms24010669	Srisongkram	Not available
10.1038/s41408-023-00798-7	Ryu	Available in Supplementary Material
10.1186/s12859-022-04662-6	Firoozbakht	Not available
10.3390/cells12141917	Ye	Not available
10.1007/s12032-022-01924-4	Ramesh	https://github.com/oddt/rfscorevs (accessed on 4 January 2026)
10.1016/j.patter.2022.100441	Pham	https://github.com/pth1993/CIGER (accessed on 4 January 2026)
10.1158/2159-8290.CD-20-1201	Carvalho	Not available
10.1093/bib/bbad441	Stourac	https://loschmidt.chemi.muni.cz/predictonco/ (accessed on 4 January 2026)
10.1016/j.csbj.2022.11.037	Zhang	https://zenodo.org/records/7385788 (accessed on 4 January 2026)
10.1186/s12859-023-05539-y	Millikin	https://github.com/stewart-lab/fast_km (accessed on 4 January 2026)
10.1186/s12859-022-05069-z	Zhang	Not available
10.3390/ijms24031878	Sun	https://github.com/2003100127/deepsmirud (accessed on 4 January 2026)
10.1038/s41598-023-50393-w	Schaduangrat	https://github.com/Shoombuatong/StackER (accessed on 4 January 2026)
10.3390/molecules27134098	Aziz	Not available
10.18632/aging.203960	Pun	Not available
10.1186/s40478-022-01463-z	Zhao	https://github.com/Bin-Chen-Lab/dipg (accessed on 4 January 2026)
